# Flavonoid intake and breast cancer risk: a case–control study in Greece

**DOI:** 10.1038/sj.bjc.6601271

**Published:** 2003-09-30

**Authors:** J Peterson, P Lagiou, E Samoli, A Lagiou, K Katsouyanni, C La Vecchia, J Dwyer, D Trichopoulos

**Affiliations:** 1 Schools of Nutrition and Medicine, Boston, MA 02111, USA; 2 Jean Mayer USDA Human Nutrition Research Center on Aging at Tufts University, Boston, MA 02111, USA; 3 Frances Stern Nutrition Center Tufts-New England Medical Center, Box 783 NEMCH 750 Washington St, Boston, MA 02111, USA; 4 Department of Hygiene and Epidemiology, School of Medicine, University of Athens, Greece, 75 M. Asias St, Goudi, GR-115 27 Athens, Greece; 5 Faculty of Health Professions, Athens Technological Institute (TEI), Greece; 6 Laboratory of Epidemiology, Mario Negri Institute Via Eritrea, 62- 20157, Milan, Italy; 7 Institute of Medical Statistics, University of Milan, via Venezian 1, 20133 Milan, Italy; 8 Department of Epidemiology, Harvard School of Public Health, 677 Huntington Avenue, Boston, MA 02115, USA

**Keywords:** flavonoids, breast cancer, flavones, case–control, antioxidants, diet

## Abstract

Flavonoids have been investigated for possible inverse associations with various chronic degenerative diseases, but there are no epidemiologic data concerning a possible association between several of the main flavonoid categories and breast cancer risk. We have applied recently published data on the flavonoid content of several foods and beverages on dietary information collected in the context of a large case–control study of 820 women with breast cancer and 1548 control women, conducted in Greece. We found a strong, statistically significant inverse association of flavone intake with breast cancer. The odds ratio for an increment equal to one standard deviation of daily flavone intake (i.e. 0.5 mg day^−1^) was 0.87, with 95% confidence interval 0.77–0.97. The association persisted after controlling for fruit and vegetable consumption, or for other flavonoid intake. This inverse association is compatible with and may explain the reported inverse association of breast cancer with consumption of vegetables, particularly leafy vegetables. After controlling for dietary confounding, there was no association of breast cancer risk with flavanones, flavan-3-ols, flavonols, anthocyanidins or isoflavones.

Flavonoids are naturally occurring substances in plants (Peterson and Dwyer, 1998). For five categories of flavonoids, namely flavones, flavonols, flavan-3-ols, flavanones and anthocyanidins, food composition data have been recently published by the US Department of Agriculture (US Department of Agriculture, 2003), while for a sixth category, that of isoflavones, food composition data have been available for some time (US Department of Agriculture-Iowa State University, 2002). Flavones, such as apigenin and luteolin, are present chiefly in grains, leafy vegetables and herbs. Flavonols are present in many plant foods: they include the glycosides of quercetin in fruits, leaves and vegetables; kaempferol in many fruits and leafy vegetables; isorhamnetin in onions and pears; and myricetin in berries, maize and tea. Flavan-3-ols, specifically the catechines, are abundant in ripe fruits, leaves, tea and chocolate. The major sources of the flavanone class in foods are citrus fruits and juices. The anthocyanidins are most abundant in fruits and less frequent in cereals and vegetables. The isoflavones include the compounds daidzein and genistein and are found mainly in soya and soya products (Peterson and Dwyer, 1998).

As several flavonoids have antioxidant properties, as well as antimutagenic and antiproliferative properties *in vitro* (Kandaswami *et al*, 1992; Franke *et al*, 1998; Takahashi *et al*, 1998; Le Marchand *et al*, 2000), these compounds have been investigated for possible inverse associations with various chronic diseases, including cardiovascular diseases and several forms of cancer. Studies have suggested that flavonoid intake may be associated with reduced risks of certain types of cancer (Stoner and Mukhtar, 1995; Barnes *et al*, 1996) and coronary heart disease (Hertog *et al*, 1995; Hollman *et al*, 1996). A special interest on breast cancer stems from the fact that several flavonoids, particularly isoflavones, have also antioestrogenic effects (So *et al*, 1997; Papas, 1999). Some (Ingram *et al* 1997; Zheng *et al*, 1999; Murkies *et al*, 2000; Dai *et al*, 2002) but not all (den Tonkelaar *et al*, 2001) studies have found that diets high in isoflavones are associated with decreased breast cancer risk. All these studies have relied on urinary excretion measurements, which can theoretically be affected by disease status. We have found no published epidemiologic data concerning a possible association between intake of any of the indicated other categories of flavonoids and breast cancer risk.

The objective of the present investigation was to ascertain whether one or more of the studied flavonoid categories was associated with breast cancer risk. For this purpose, we applied recently published data on the flavonoid content of several foods and beverages (US Department of Agriculture-Iowa State University, 2002; US Department of Agriculture, 2003) on dietary information collected in the context of a large case–control study of breast cancer, conducted in Greece in the early 1990 s. In that study (Katsouyanni *et al*, 1994; Trichopoulou *et al*, 1995), no association was found between intake of energy-generating nutrients and breast cancer risk, but consumption of fruits and vegetables was inversely associated with the risk.

## SUBJECTS AND METHODS

This study relies on data from a case–control study on diet and breast cancer risk undertaken in Athens, Greece, from 1989 to 1991. The study included 820 incident histologically confirmed cases of breast cancer, hospitalised in four major hospitals in the Greater Athens area, and 1548 control women, who were either hospital visitors or orthopaedic patients. Cases and controls were interviewed in the hospitals by trained interviewers. The interview covered demographic and reproductive information, and included a semiquantitative food frequency questionnaire. An expanded version of this questionnaire has been subsequently validated (Gnardellis *et al*, 1995). Details about the study, case eligibility and control selection procedures, as well as results concerning food groups and energy-generating nutrients in relation to breast cancer risk have been previously reported (Katsouyanni *et al*, 1994; Trichopoulou *et al*, 1995).

All subjects were asked to indicate the average frequency of consumption per month, per week or per day, of 115 food items or beverage categories, during a period of 1 year before onset of the present disease or before the interview for visitor controls. Frequencies were translated into quantities using typical portions sizes for each food item or beverage category (Katsouyanni *et al*, 1994) and quantities were converted into average daily intakes of various categories of flavonoids. Food and beverage contents of flavones, flavonols, flavan-3-ols, flavanones, anthocyanidines and isoflavones were obtained from the US Department of Agriculture databases (US Department of Agriculture-Iowa State University Database, 2002; US Department of Agriculture, 2003).

The data were modelled through logistic regression (Breslow and Day, 1980) using the SPSS statistical package (Statistical Package for Social Sciences v. 11.5, Chicago, IL, USA). The effects of several established demographic and reproductive risk factors for breast cancer were evident in the present study (Katsouyanni *et al*, 1994). Thus, a core model was used that included age (in years, continuously), place of birth (urban vs rural), body mass index (in kg m^−2^, continuously), parity (parous vs nulliparous), age at first pregnancy (in years, continuously, among parous women), age at menarche (in years, continuously), menopausal status (post- vs premenopausal) and ethanol intake (<1 glass per month vs ⩾1 glass per month). Exogenous oestrogens were infrequently used in Greece in the late 1980s, as reflected also in our study sample; therefore, exogenous oestrogen use was not controlled for in this analysis (Lipworth *et al*, 1995). As intake of most food items, food groups and nutrients is positively associated with total energy intake, the latter variable was always adjusted for in the evaluation of the association between flavonoid intake and breast cancer risk (Willett and Stampfer, 1998). Breast cancer odds ratios (ORs) were expressed per increments equal to 1 s.d. each of the evaluated categories of flavonoids.

## RESULTS

Table 1

**Table 1:** Basic demographic, lifestyle and reproductive characteristics of 820 women with breast cancer and 1548 control women

	**Cases^b^,^c^**	**Controls^b^,^c^**
Age (years)	56.4 (0.43)	54.4 (0.32)
*Place of birth*		
Urban	620 (75.7)	1106 (71.6)
Rural	199 (24.3)	439 (28.4)
Body mass index (kg m^−2^)	26.6 (1.02)	25.9 (0.75)
Parity		
Parous	657 (80.2)	1164 (75.2)
Nonparous	162 (19.8)	384 (24.8)
Age at first birth (years)	26.4 (0.21)	25.9 (0.16)
Age at menarche (years)	12.9 (0.06)	13.1 (0.04)
*Menopausal status*		
Postmenopausal	550 (67.1)	1041 (67.3)
Premenopausal	270 (32.9)	505 (32.7)
*Alcohol consumption*		
⩾1 glass per month	591 (72.2)	1086 (70.2)
<1 glass per month	228 (27.8)	462 (29.8)

a Adapted from Katsouyanni *et al* (1994).

b Nonadditivity is accounted for by a few missing values.

c In parentheses: standard errors for quantative variables; percentages for categorical variables.

shows the basic demographic and reproductive characteristics, as well as patterns of alcohol consumption of 820 women with incident breast cancer and 1548 control women. These data serve only descriptive purposes, because cases are older than controls and the indicated variables are not mutually adjusted for.

In Table 2

**Table 2:** Distribution of 820 women with breast cancer and 1548 control women by marginal frequency of consumption of food group sources of flavonoids

	**Quintile^b^**					
	**1 (lowest)**	**2**	**3**	**4**	**5 (highest)**	***X***^c^ **linear trend**
*Cereals*						
Cases	164	146	164	176	169	1.78
Controls	369	270	309	297	303	
Quintile median (times per month)	33	39	66	97	138	
*Vegetables*						
Cases	190	174	153	162	140	−2.58
Controls	293	301	309	315	330	
Quintile median (times per month)	47	74	90	108	142	
*Starchy roots*						
Cases	244	263	188	124	—	1.42
Controls	448	545	348	207		
Quintile median (times per month)	4	8	8	16		
*Pulses, nuts and seeds*						
Cases	184	180	136	134	185	2.55
Controls	391	336	290	244	287	
Quintile median (times per month)	2	4	5	7	13	
*Fruits*						
Cases	168	180	171	155	145	−1.63
Controls	308	295	302	318	324	
Quintile median (times per month)	43	78	109	137	183	
*Energy Intake*						
Cases	150	167	164	159	176	2.03
Controls	322	305	309	313	296	
Quintile median (kJ day^−1^)	5693	6855	7793	8809	10 583	

a Adapted from Trichopoulou *et al* (1995).

b Nonadditivity is accounted for by a few missing values.

c *X* values are age-adjusted and are interpretable as standard normal deviates.

, cases and controls are distributed by marginal case and control-combined quintiles of total energy intake and food groups that are known major sources of flavonoids in the diet. Age-adjusted linear trends assessing the difference in the distributions of the indicated food groups and energy intake between cases and controls are also shown. Linear trends were assessed through *X*, which is the square root of *χ*^2^ with one degree of freedom, as suggested by Armitage (1955) and generalised over several strata by Mantel (1963). Cases reported higher intakes of cereals, starchy roots, pulses and total energy, and lower intakes of vegetables and fruits. Since comparisons are neither energy nor mutually adjusted among flavonoid categories, valid differences cannot be drawn from these data.

In Table 3

**Table 3:** Distribution of 820 women with breast cancer and 1548 control women by marginal quintiles of flavonoid intake categories

	**Quintile^a^**					
	**1 (lowest)**	**2**	**3**	**4**	**5 (highest)**	***X***^b^ **linear trend**
*Flavanones*						
Cases	162	167	158	178	154	0.29
Controls	311	307	315	296	319	
Quintile median (mg day^−1^)	9.1	20.3	33.5	44.4	67.1	
*Flavan-3-ols*						
Cases	169	174	170	158	140	−2.10
Controls	300	295	300	311	329	
Quintile median (mg day^−1^)	9.0	16.0	23.5	30.7	45.2	
*Flavonols*						
Cases	179	167	153	159	151	−1.65
Controls	289	302	315	310	317	
Quintile median (mg day^−1^)	9.7	15.9	19.4	24.1	30.6	
*Flavones*						
Cases	197	187	158	146	131	−4.63
Controls	276	287	315	328	342	
Quintile median (mg day^−1^)	0.3	0.3	0.4	0.7	1.1	
						
*Anthocyanidins*						
Cases	186	251	55	170	149	−0.83
Controls	319	459	157	286	315	
Quintile median (mg day^−1^)	5.1	12.0	20.9	40.7	81.4	
*Isoflavones*						
Cases	172	149	174	158	166	0.71
Controls	301	329	295	316	307	
Quintile median (mg day^−1^)	0.01	0.2	0.2	0.3	0.8	

a Nonadditivity is accounted for by a few missing values.

b *X* values are age-adjusted and are interpretable as standard normal deviates.

, cases and controls are distributed by the studied categories of flavonoids in the diet. There is evidence that breast cancer risk is inversely associated with flavone intake and less strong evidence for inverse associations with flavan-3-ol and flavonol intake. However, as in Table 2, the data in Table 3 are neither energy nor mutually adjusted and the patterns are not directly interpretable.

In Table 4

**Table 4:** Multiple logistic regression-derived ORs for breast cancer, per 1 s.d. increment of each of the examined flavonoid categories

**Flavonoid category**	**Odds ratio**	**95% confidence interval**	***P*****-value**
*Flavanones (per 24.3 mgday*^−*1*^*)*			
Model I^a^	0.92	0.83–1.02	0.09
Model II^b^	1.13	0.98–1.29	0.09
Model III^c^	0.96	0.87–1.07	0.44
*Flavan-3-ols (per 16.2 mg day*^−*1*^*)*			
Model I^a^	0.83	0.75–0.93	0.001
Model II^b^	0.97	0.83–1.14	0.71
Model III^c^	0.93	0.78–1.11	0.43
*Flavonols (per 8.3 mg day*^−*1*^*)*	0.81	0.73–0.90	0.001
Model I^a^	0.93	0.81–1.08	0.35
Model II^b^	0.91	0.78–1.06	0.22
Model III^c^			
*Flavones (per 0.5 mg day*^−*1*^*)*	0.84	0.75–0.93	0.001
Model I^a^	0.86	0.77–0.96	0.01
Model II^b^	0.87	0.77–0.97	0.02
Model III^c^			
*Anthocyanidins (per 50.0 mg day*^−*1*^*)*			
Model I^a^	0.86	0.76–0.97	0.01
Model II^b^	0.95	0.83–1.08	0.40
Model III^c^	0.94	0.81–1.09	0.39
*Isoflavones (per 0.8 mg day*^−*1*^*)*			
Model I^a^	1.07	0.97–1.18	0.15
Model II^b^	1.05	0.95–1.16	0.31
Model III^c^	1.07	0.97–1.18	0.17

a Adjusted for age, place of birth, parity, age at first pregnancy, age at menarche, menopausal status, body mass index, total energy intake and alcohol consumption.

b Adjusted as in model I, controlling also for fruit and vegetable consumption.

c Adjusted as in model I, and mutually between flavonoid categories.

, OR (and 95% confidence intervals) of breast cancer for 1 s.d. increase in the consumption of each of the examined major categories of flavonoids are presented. For each of the six categories of flavonoids, ORs derived from three different models are shown. In model I, the OR is adjusted for the sociodemographic, lifestyle and reproductive variables shown in Table 1, as well as for energy intake. These OR estimates may be confounded by the intake of other flavonoid categories or by other compounds in vegetables and fruits that are inversely associated with breast cancer risk in these data. In model II, the ORs are adjusted for fruit and vegetable consumption, in addition to the variables controlled for in model I. In model III, the ORs for each category of flavonoids are adjusted mutually, as well as for the variables included in model I. Thus, OR estimates from models II and III are less subject to confounding by other compounds in fruits and vegetables, and other flavonoids in the diet. It is apparent that the OR for flavones is fairly robust and indicates a statistically significant inverse association with breast cancer risk, even after taking into account the potential confounding effect of fruit, vegetable and other flavonoid intake. No such evidence exists for any other category of flavonoids examined.

## DISCUSSION

We have found evidence that flavones are inversely related to breast cancer risk. The inverse association of flavone intake with breast cancer was only marginally affected when intake of fruits and vegetables, or other flavonoids was accounted for. The inverse association of flavones with breast cancer is not trivial, since it implies a 13% reduction in breast cancer risk per 1 s.d. (0.5 mg day^−1^) of increase in the intake of the respective compounds. Inverse associations with breast cancer risk were also found for flavonols, flavan-3-ols and anthocyanidins. These associations were sharply attenuated and became nonsignificant, however, when intake of fruits and vegetables or other flavonoids were controlled for. We found no evidence that flavanones had a major effect on breast cancer risk and, for isoflavones, the evidence, if any, was for a positive rather than inverse association.

Very few studies have examined flavonoids in relation to breast cancer risk. No association between cancer, including breast cancer, and total flavonoids was found in the combined analysis of the 16 cohorts of the Seven Countries Study (Hertog *et al*, 1995). An inverse association between urinary excretion of phyto-oestrogens, including isoflavones, was found among Chinese women in Shanghai (Zheng *et al*, 1999; Dai *et al*, 2002) and Australian women (Ingram *et al*, 1997; Murkies *et al*, 2000), but no such association was evident in a similar study in Netherlands (den Tonkelaar *et al*, 2001). With respect to isoflavones, our data do not support those reported from studies in China and Australia. There are several possible explanations: intake of soya and soya products has been, and still is, very limited in Greece; an inverse association between isoflavones and breast cancer risk may not be captured through a dietary intake study, but may be ascertained in studies employing measurements of urinary excretion; or reduced urinary excretion of isoflavones may be a consequence rather than cause of breast cancer and the procedures associated with the diagnosis and treatment of this disease. There are no comparable data in the literature concerning flavone intake in relation to breast cancer risk so, at this stage, our findings concerning these compounds should be considered as hypothesis generating rather than as documenting a genuine association.

As flavones are largely derived from grains and vegetables (Peterson and Dwyer, 1998), and there is no evidence in the literature that grains or cereals are inversely associated with breast cancer risk (World Cancer Research Fund and American Institute for Cancer Prevention, 1997), our findings point to leafy vegetables and herbs as the food groups with potential beneficial properties for breast cancer risk. A source of concern is that vegetables were more strongly inversely associated with breast cancer risk in this Greek study (Trichopoulou *et al*, 1995) than in other case–control and particularly cohort investigations (World Cancer Research Fund and American Institute for Cancer Prevention, 1997; Smith-Warner *et al*, 2001; Lagiou *et al*, 2002). However, consumption of vegetables and variability of consumption is higher in the Greek population than in most other populations (Agudo *et al*, 2002) and Greek food patterns are characterised by high consumption of wild greens that are rich in flavones (Trichopoulou *et al*, 2001).

Strengths of this study are its relatively large study size, the use of a validated food frequency questionnaire and the reliance on generally sound food composition databases (US Department of Agriculture-Iowa State University Database, 2002; US Department of Agriculture, 2003). Limitations of the study are the lack of a flavone-specific prior hypothesis, the emergence of findings after undertaking multiple analyses and questions concerning the applicability of US-based flavonoid food composition tables to Greek foods. A generic limitation is that confounding by dietary factors that have not been measured cannot be controlled for (Davey Smith and Ebrahim, 2003).

Various categories of flavonoids have been reported to inhibit breast cancer cell replication, oestrone sulphatase activity and mammary gland tumorigenesis in experimental analyses (So *et al*, 1996; Huang *et al*, 1997; Kuntz *et al*, 1999). However, except with respect to isoflavones, there is no sufficient evidence, experimental or otherwise, linking particular flavonoid compounds or categories to specific actions in the process of mammary carcinogenesis. Consequently, the biological plausibility of an inverse association between flavones and breast cancer risk can, at this stage, be considered as no more than suggestive.

In conclusion, we have found evidence that intake of flavones – but not intake of flavonols, flavan-3-ols, flavanones or anthocyanidins or isoflavones – may be inversely related to breast cancer risk. This inverse association is compatible with and may explain the reported inverse association of breast cancer with consumption of vegetables.
